# Global patterns of plumage color evolution in island-living passeriform birds

**DOI:** 10.1371/journal.pone.0294338

**Published:** 2023-12-15

**Authors:** Madison D. Oud, Sean M. Mahoney, Claudie Pageau, Marcio Argollo de Menezes, Nathan Smith, James V. Briskie, Matthew W. Reudink

**Affiliations:** 1 Department of Biological Sciences, Thompson Rivers University, Kamloops, BC, Canada; 2 School of Natural Resources and the Environment, The University of Arizona, Tucson, AZ, United States of America; 3 Physics Institute, Fluminense Federal University, Niteroi, Brazil; 4 National Institute of Science and Technology on Complex Systems, Rio de Janeiro, Brazil; 5 School of Biological Sciences, University of Canterbury, Christchurch, New Zealand; Oklahoma State University, UNITED STATES

## Abstract

Island environments have the potential to change evolutionary trajectories of morphological traits in species relative to their mainland counterparts due to habitat and resource differences, or by reductions in the intensity of social or sexual selection. Latitude, island size, and isolation may further influence trait evolution through biases in colonization rates. We used a global dataset of passerine plumage color as a model group to identify selective pressures driving morphological evolution of island animals using phylogenetically-controlled analyses. We calculated chromaticity values from red and blue scores extracted from images of the majority of Passeriformes and tested these against the factors hypothesized to influence color evolution. In contrast to predictions based on sexual and social selection theory, we found consistent changes in island female color (lower red and higher blue chromaticity), but no change in males. Instead, island size and distance from mainland and other islands influenced color in both sexes, reinforcing the importance of island physiognomy in shaping evolutionary processes. Interactions between ecological factors and latitude also consistently influenced color for both sexes, supporting a latitudinal gradient hypothesis. Finally, patterns of color evolution varied among families, indicating taxon-specific microevolutionary processes in driving color evolution. Our results show island residency influences color evolution differently between sexes, but the patterns in both sexes are tempered by ecological, island characteristics, and phylogenetic effects that further vary in their importance among families. The key role of environmental factors in shaping bird plumage on islands further suggests a reduced importance of sexual and social factors in driving color evolution.

## Introduction

The trajectory of trait evolution of species living on islands often differs from that of their mainland counterparts, and this has been attributed to differences in habitat and resources, or by changes in the intensity of social or sexual selection [1]. Compared to continental habitats, islands are isolated and relatively small, but are replicated repeatedly across a broad geographic scale, making them ideal systems to study the evolutionary processes that shape variation in traits [2]. A number of “island syndrome” studies have documented the parallel evolution of island species when compared to mainland populations [3,4]. According to the “island rule” [1], body sizes in large vertebrates trend towards dwarfism while small vertebrates trend towards gigantism when comparing island to mainland populations [4–7]. Island vertebrates also tend to exhibit K-selected life history strategies, as evidenced by relatively low fecundity, longer developmental periods, and high survival [5,8]. Typically, island rule studies focused on body size and life history, while the impact of island environments on other traits, such as ornamental traits, remains less understood.

Animal color is an important and complex signal used in both inter- and intraspecific interactions [9] and is thought to evolve in response to a variety of evolutionary mechanisms including natural selection, sexual selection, genetic drift, environmental conditions, arbitrary mate choice [10], or some combination of these factors [11]. In birds, plumage coloration varies widely within and among species [12,13], and color signals may play important evolutionary roles by mediating mate choice, species recognition, and predator avoidance [14]. As such, there is considerable interest in understanding the factors driving global patterns of plumage color [15–20].

Analyses of plumage coloration reveal a general pattern of dull coloration in island birds, but most of these studies are constrained by small geographic scope or use of relatively few species [16,17,19], but see [15]. A recent worldwide analysis [20] compared plumage coloration of 116 island species to closely related mainland species and found a reduction in plumage brightness and color intensity as well as a reduction in the number of color patches in island species. Another large-scale study [21] found color differences between mainland and island birds in 731 species and subspecies across three families (Meliphagidae, Fringillidae, and Monarchidae), but the direction of this effect was complex and varied by family: Meliphagidae shifted towards melanin-based plumage while Fringillidae shifted away from carotenoid plumage on island environments. Together, these results suggest different selective pressures may be operating in different lineages or vary geographically. However, these studies collectively represent <10% of passerine birds, and whether the patterns of trait evolution are generalizable to other taxa, and even other passerine families, remains to be tested.

Several hypotheses have been proposed to explain color loss in island birds. If plumage color functions in interspecific interactions, island birds may be duller colored due to reduced selection for species recognition as island systems typically contain fewer sympatric species than continental areas [16,17]. Alternatively, if exaggerated color expression is under condition-dependent sexual selection [22], then island species may become less colorful because of reduced sexual selection pressure on islands [17,23]. Sexual selection is predicted to be relaxed on islands because of reduced genetic diversity from founder effects [24] and/or reduced parasite pressure [25], diminishing the indirect fitness benefits from extra-pair copulations [26]. The idea of reduced sexual selection pressure on islands is supported by lower extra-pair paternity rates in island species [27]. Changes in the costs of bright plumage may also vary between island and continental habitats. For example, predation pressure on islands is often lower [28] and thus could promote elaboration of plumage coloration rather than camouflage [29,30]. Island species may also show decreased territoriality in part due to fewer con- and heterospecifics, relaxed sexual selection pressure, and/or increased resource availability [20], possibly reducing the need to signal territoriality during species interactions [31]. Food resources on islands may differ from those on the mainland, and carotenoid-deficient diets may reduce carotenoid-based (red, orange, yellow) plumage expression [22]. Some island taxa do not follow predicted patterns of plumage color evolution possibly resulting from repeated founder effects [32,33].

Assessing color variation in island birds can be challenging due to confounding ecological and natural history factors. First, color evolution may be affected by latitudinal differences among species. Known as Gloger’s Rule, this biogeographic rule predicts animal coloration will covary with latitudinal changes in body temperature regulation, colors needed for camouflage, parasite loads, or some combination of these factors [34]. Second, differences in habitat may influence ambient light in the environment, so there may be selection for or against ornate plumage given habitat-specific light conditions [35]. Third, island characteristics such as geographic size and isolation (i.e., distance from mainland) may affect the evolutionary trajectory of color evolution by biasing colonization or by limiting population sizes, thereby diminishing or exacerbating genetic drift effects [24]. Finally, macroevolutionary studies often assess higher-level taxonomic processes, but because selection pressures likely vary among families, the directionality of the effects may change at lower taxonomic levels [36,37]. To date, no study has comprehensively tested the combination of these social and ecological factors influencing bird coloration. To unravel the mechanisms mediating plumage evolution, it is important to assess the biological and ecological factors contributing to color at a global scale and across a broad range of taxa.

Although previous studies have documented color differences between mainland and island bird populations using a few families or subsets of species (e.g., [20,21], no study has comprehensively assessed the selective pressures driving plumage color evolution in island birds using an entire order of birds while also simultaneously controlling for confounding biotic and abiotic factors, calling into question the generalizability of an island effect on plumage coloration. Additionally, few studies simultaneously test color evolution in both sexes [38] despite growing interest in female communication trait evolution [39]. Using a phylogenetic statistical framework, we leveraged a global and comprehensive dataset of plumage color in the Order Passeriformes to test the hypothesis that color would differ between mainland and island species. We specifically tested the prediction that male and female passerines occupying islands would be less colorful than those occupying the mainland (*sensu* [20], while also controlling for covariates such as latitude (i.e., “Gloger’s Rule” [34]), diet [11], and habitat, as a proxy of the light environment [35]. Given that selection pressures may vary among species within Passeriformes, we also tested for color differences between mainland and islands species at the family-level. Finally, because island size and island isolation may influence species richness, resource availability, predation pressure, and/or bias island colonization (*sensu* [20,30], we also tested the effect of island size and isolation on plumage coloration.

## Materials and methods

The Passeriformes provide an ideal model to test hypotheses on color evolution because 1) it is a speciose order (more than half of all living birds), 2) passeriform species generally exhibit relatively ornate plumage coloration [15], 3) there is high variation in plumage color among species, and 4) passeriform birds are broadly distributed throughout the world in both mainland and island habitats [40].

### Data collection

**Fig 1:**
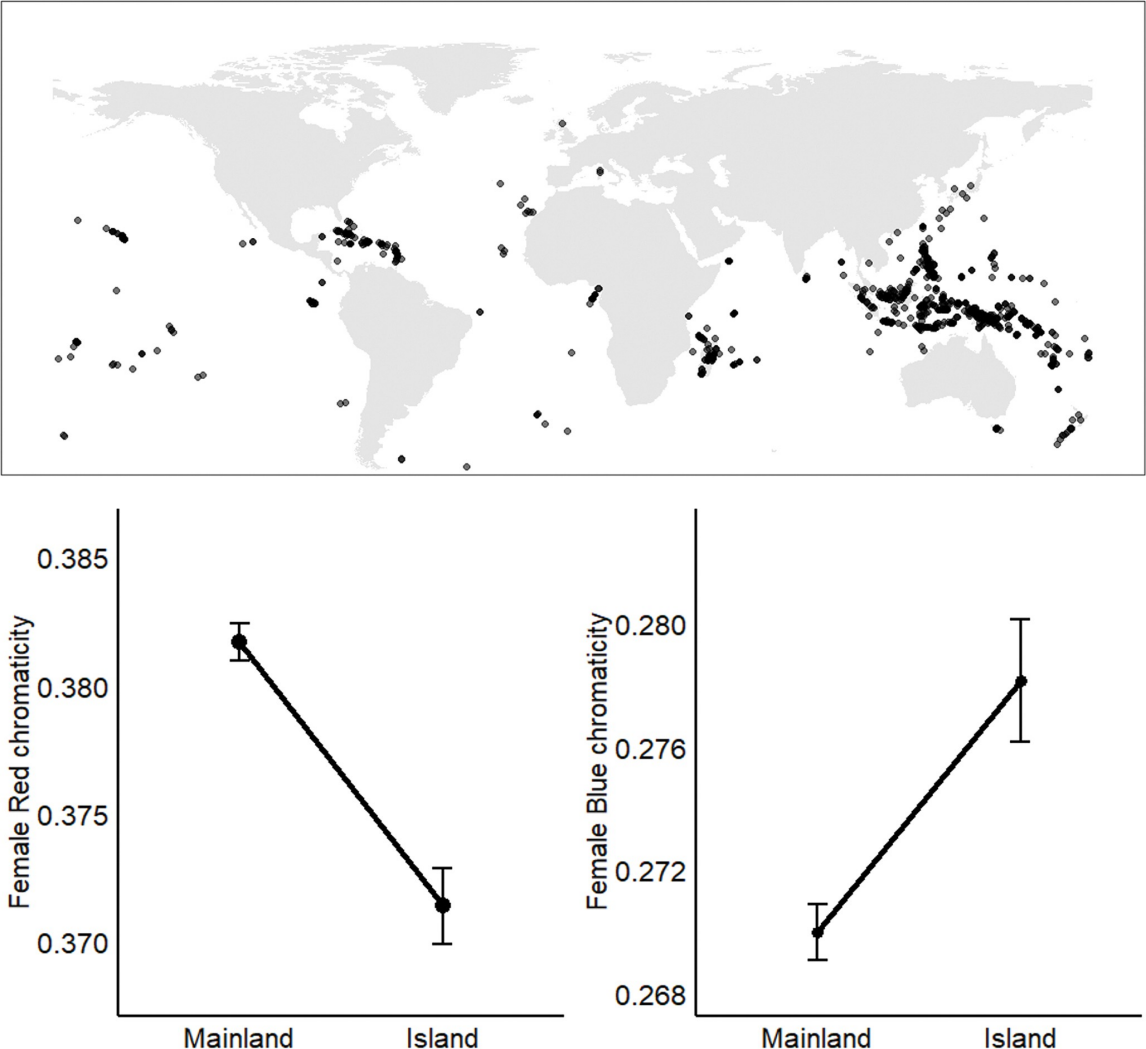
Islands are useful systems to study plumage coloration and the color of island species differs from their mainland counterparts. Top panel: Islands are isolated, relatively small, and are replicated across a broad geographic scale, making them ideal systems to study evolutionary processes of trait variation. Dots represent global distribution of Passeriformes island species (n = 1,183) in our study. Bottom panels: Female colour variation between mainland and island passerine birds (n = 5,693). After controlling for phylogeny and ecological covariates, red chromaticity (±SE) was significantly higher (F = 57.2, P<0.0001) and blue chromaticity (±SE) was significantly lower on mainlands (F = 23.7, P<0.0001) relative to islands. Means from raw data are shown. Map was generated using the sf package [48] in R.

We classified 5,693 Passeriformes species (S1 Dataset) as mainland or island dwelling (Fig 1) using global range maps from the International Union of Conservation of Nature’s Red List of Threatened Species [41]. As in similar studies, we defined islands as land masses smaller or equal to 785,753 km^2^ (the island of Papua, Indonesia and Papua New Guinea [40,42]. All other land masses were categorized as mainlands. Passerines where >80% of their range covered non-continental landmasses (such as the Hawaiian islands or New Zealand) were classified as “island” species (n = 1287). Species were classified as “mainland” when >80% or more of the range covered a continent (such as North America or Australia) (n = 4406). Throughout, we use the term “land classification” to refer to designation of a species as either an island or continental species. Using IUCN range extents, we also calculated the latitude centroid of each species range. Diet, habitat type, and geographic region for each species were taken from [43] based on [44–46]. These factors may influence plumage coloration differences between island and continental species because of variation in pressures associated with thermoregulation [47], resource availability, dietary precursors [20], or light environments [35]. We obtained data on island size, distance to nearest mainland, and distance to nearest other islands from the UN Environmental Programme island directory [42]. Island size may influence color evolution because there may be a greater diversity of predators on larger islands [30], while a lower diversity of congeneric species on smaller islands may reduce species recognition pressures [20]. Distance to continents and other islands may influence the evolutionary trajectory of coloration of island birds through founder effects of relatively few individuals and alleles [40]. Collectively, our dataset included land classification (island vs. continental), habitat, diet, geographic region, and range latitude for passerines, as well as island size, distance to mainland, and distance to other islands for passerines found on islands (Supporting Information).

Taxonomic relationships are in constant revision [49]. As such, including all island and continental species and subspecies was not possible as the phylogenetic relationships among all taxa are not fully resolved and ecological data were only available for some. To solve this problem, we restricted our analyses to species with color scores from [38], taxa included in BirdTree [50,51], a widely used taxonomy in studies of avian evolution (e.g. [52–54]), and species for which we able to obtain sufficient ecological and geographic data. In polytypic species, we used the nominate subspecies consistent with [38,49–51]. [38] quantified data for 5,831 Passeriformes species, but we were unable to obtain sufficient information on coloration, ecology, geography, and/or phylogenetics [50,51] for n = 138 species. Of the missing species, n = 35 were island species, representing 0.6% of our dataset (Supporting Information). Subsequently, the data deficient taxa were not included in our analyses, resulting in a 1% decrease in species used by [38]. Such a decrease in taxon sampling approximates that of other work on avian morphology evolution [49] (see Statistical Analysis section for more details).

Next, we quantified plumage coloration for passeriform species. Typically, studies of plumage ornamentation employ reflectance spectrometry as an objective measure that quantifies plumage coloration and accounts for birds’ visual system [55]. While this approach is suited to intraspecific studies to capture within-species variation (e.g., [56]), or studies using a smaller subset of species comparisons (e.g., [20]), it poses a challenge in large-scale analyses, particularly because interspecific variation in plumage elaboration means that the appropriate plumage patch(es) for scans can differ from one taxa to another. Instead, an approach that captures the extent of elaboration in a single metric is better suited for large-scale interspecific comparisons, such as the color scores developed by [38]. Thus for our study, we used plumage color data from [38], who quantified red, green, and blue (RGB) values from images of the crown, forehead, nape, throat, upper breast and lower breast of the Passeriformes species listed in the Birds of the World [57]. [38] developed this approach specifically to quantify the degree to which a particular plumage is “male-like”, allowing for large-scale interspecific comparative studies, using a single metric that captures the degree of elaboration in plumage ornamentation. Importantly, [38] verified their color scores were consistent with estimates from spectrometry on museum specimens (R^2^ = 0.67, P<0.0001, see their Extended Data Fig 1 and Extended Data Plumage scores validation analysis section in [38]. [38] used these scores to explore correlated evolution between sexes and the effects of morphological, social, and life-hisotry traits in the evolution of color elaboration. As the authors conclude, these color scores are ideal for hypothesis testing on the function and evolution of color ornamentation in both males and females—just as we do here. One constraint of the [38] color scores is that all forms of elaboration are treated in the same way (i.e., elaboration of carotenoid- and structural-based colors are considered similarly). However, because carotenoid- and structural-based coloration arise through different physiological mechanisms, we used color scores extracted from the RGB values [38] to calculate chromaticity. This approach is particularly useful for our purposes, as high red chromaticity are associated with carotenoid pigmentation [58–60]. Red chromaticity was calculated using the equation: R/(R+G+B), and blue chromaticity was calculated using the equation: B/(R+G+B), where R is the red value, G is the green value, and B is the blue value from [38]. We previously used chromaticity scores to evaluate the evolutionary mechanisms of Tyrannidae plumage coloration [61].

Although chromaticity values provide a metric to quantify the relative short and long wavelengths and can reliabily reflect carotenoid and structural plumage coloration [60], to further assess the efficacy of using chromaticity to estimate “carotenoid” and “structural” plumage coloration, a single, independent observer categorized the color for multiple plumage patches in males and females of all species in the Family Thraupidae (n = 346 species/sex) using the publicly available visual media source Birds of the World by the Cornell Lab of Ornithology [62]. In this analysis, we used 10 patches: auricular, crown, back, rump, throat, breast, belly, crissum, wingtip (color of the tip of the longest primary flight feather), and dorsal side of the rectrices. Patches were chosen to mirror [63], who measured the reflectance spectrum of each patch color using a spectrophotometer for most members in Thraupidae. We then compared the chromaticity values to these classifications and found the distribution of red and blue chromaticity scores did not overlap in plumage patches classified as “blue” or “red.” This suggests that our chromaticity values effectively captured the variation in structural blue and carotenoid red plumage coloration (S1 Fig in S1 File).

### Phylogenetic methods

To control for phylogenetic relationships in our analyses, we downloaded 1,000 potential phylogenies from birdtree.org [50,51] for the 5,693 passerine species included in the dataset. We used TreeAnnotator in BEAST v1.10.1 [64] to construct a maximum clade credibility tree using 1% burn in and mean node heights. We repeated these steps with the 1,183 island passerines to test the effect of island characteristics on passeriform color.

### Statistical analysis

We performed all analyses in R 3.5.3 [65] using phylogenetic generalized least squares (PGLS) in the *nlme* package [66]. We tested how male and female passeriform color variation was explained by land classification, diet, habitat, latitude, and region using stepwise model reduction based on Akaike Information Criterion (AIC). We first built a full model, which included either red or blue chromaticity as the response variable, and land classification, diet, habitat, latitude, region, and their interactions (land classification x latitude, land classification x diet, land classification x habitat, and habitat x diet) as the main effects. We built separate models for each sex. We then undertook model reduction for all possible models using the *StepAIC* function in the *MASS* package and selected the top model based on the change in AIC (ΔAIC, [67]) between the full model and each reduced model. We considered ΔAIC values within 4 to be competitive and chose our final model based on the lowest AIC [67]. To assess differences in directionality among families, we included family as a fixed effect in the final model and plotted the results for each family. We then repeated these steps using only island passerines and included island size, distance to mainland, and distance to other islands and two-way interactions between all terms. We used phylogenetic path analyses using the R package *phylopath* to assess the direct and indirect effects of the variables from the top PGLS models. We first built candidate path analyses informed by the PGLS models and then ranked models using an information theory approach based on C-statistics [68]. Information theory evaluates the conditional independencies of each model and assigns a C statistic. The models are ranked based on the change in C statistic (ΔCICc) between models, where lower C scores are optimized models and ΔCICc <4 are competitive. The top phylogenetic path analysis model was then selected as the model with lowest C statistic.

As noted, we were not able to include all species and subspecies in our analyses. Although incomplete taxon sampling may theoretically lead to inaccuracies [69], empirical studies comparing complete or incomplete taxon sampling indicate no difference in phylogenetic inference [70–72]. Studies assessing evolutionary geographic patterns similarly indicate incomplete taxon sampling does not affect results, so long as the included taxa are geographically well-represented and the phylogenetic trees are robust [73,74]. In our study, we did not include n = 138 Passeriformes species from [38] due to insufficient data (see Data Collection section above), resulting in a 1% decrease in taxon sampling. However, our data included representatives from >98% of families (138 of 140) and >99% of genera (1,169 of 1,177) in Passeriformes [50], the samples were widely distributed geographically, and our phylogenetic trees are commonly used in comparative bird morphology studies (e.g., [49,52–54]), thus meeting the criteria necessary to recover from incomplete taxon sampling [73,74].

## Results

### Effect of island residency and ecological factors on Passeriformes colour evolution

Based on our model selection (S1 Table in S1 File), we found mixed support for our prediction that island birds have reduced coloration (Table 1). Color differed significantly between mainland and island females, but not males (Fig 1). Female red chromaticity was higher on the mainland than on islands (Fig 1, F = 57.21, P<0.0001) while female blue chromaticity was lower on the mainland relative to islands (Fig 1, F = 23.72, P<0.0001).

**Table 1:** Plumage color in island birds is affected by island residency, diet, and geography.

Sex	Variable	Fixed effect	*df*	*F*	*P*
Female	Red chromaticity	**Land classification**	**1**	**57.21**	**<0.0001**
		**Habitat**	**3**	**22.40**	**<0.0001**
		**Diet**	**2**	**5.69**	**0.003**
		**Latitude**	**1**	**65.39**	**<0.0001**
		**Geographic region**	**6**	**15.54**	**<0.0001**
		**Land x Latitude**	**3**	**5.51**	**0.001**
		**Land x diet**	**2**	**10.60**	**<0.0001**
		**Habitat x diet**	**6**	**4.55**	**<0.0001**
	Blue chromaticity	**Land classification**	**1**	**23.72**	**<0.0001**
		**Habitat**	**3**	**20.09**	**<0.0001**
		Diet	2	3.00	0.05
		**Latitude**	**1**	**75.58**	**<0.0001**
		**Geographic region**	**6**	**7.31**	**<0.0001**
		Land x Latitude	1	2.54	0.11
		**Land x diet**	**2**	**17.93**	**<0.0001**
		**Habitat x diet**	**6**	**3.72**	**<0.0001**
Male	Red chromaticity	Land classification	1	0.05	0.82
		Habitat	3	1.17	0.32
		**Diet**	**2**	**22.46**	**<0.0001**
		**Latitude**	**1**	**15.81**	**<0.0001**
		**Geographic region**	**6**	**11.28**	**<0.0001**
		Land x Latitude	1	2.17	0.14
		**Land x diet**	**2**	**25.06**	**<0.0001**
		**Habitat x diet**	**6**	**2.61**	**0.02**
	Blue chromaticity	Land classification	1	2.21	0.14
		Habitat	3	1.96	0.12
		**Diet**	**2**	**30.09**	**<0.0001**
		**Latitude**	**1**	**14.01**	**<0.0001**
		**Geographic region**	**6**	**7.55**	**<0.0001**
		**Land x diet**	**2**	**21.13**	**<0.0001**
		**Habitat x diet**	**6**	**2.72**	**0.01**

AIC selected model results demonstrating the effect of each fixed effect and interactions on female and male Passeriformes plumage coloration. Significant results are indicated in bold text.

Apart from the effects of islands on plumage, we found color varied with several ecological and natural history covariates and their interactions, underscoring the complexity of the macroevolutionary processes driving plumage color evolution (Table 1). In support of Gloger’s Rule, color varied by latitude (S2 Fig in S1 File): red chromaticity was higher and blue chromaticity was lower near the equator for both males (F = 15.81, P<0.0001) and females (F = 65.39, P<0.0001). However, the interaction between land classification and latitude revealed red chromaticity in island females was positively related to latitude, while mainland red chromaticity was negatively related to latitude (S2 Fig in S1 File, F = 5.51, P = 0.001).

Color also varied with diet, where both invertivore and omnivore guilds had higher red chromaticity (female: F = 5.69, P = 0.003; male: F = 22.46, P<0.0001) and blue chromaticity (Table 1, male: F = 30.09, P<0.0001). However, the interactions between diet guild and land classification indicated that red chromaticity in invertivores was lower on islands (female: F = 10.60, P<0.0001; male: F = 25.06, P<0.0001). Blue chromaticity in invertivores and herbivores was higher on islands but did not vary between islands and mainlands for omnivores (S3 Fig in S1 File, female: F = 17.93, P<0.0001; male: F = 21.13, P<0.0001).

Color varied among habitat types in females, with species in open habitats having higher red chromaticity (F = 22.40, P<0.0001) while those in dense habitats had lower blue chromaticity (Table 1, F = 20.09, P<0.0001). The interaction between habitat and guild indicates red chromaticity in herbivorous birds was higher in dense and open habitats, but lower in aquatic habitats (F = 4.55, P<0.0001). Similarly, blue chromaticity in herbivorous birds was lower in dense and open habitats but higher in aquatic habitats (Table 1, F = 3.72, P<0.0001).

### Phylogenetic path analyses

**Fig 2:**
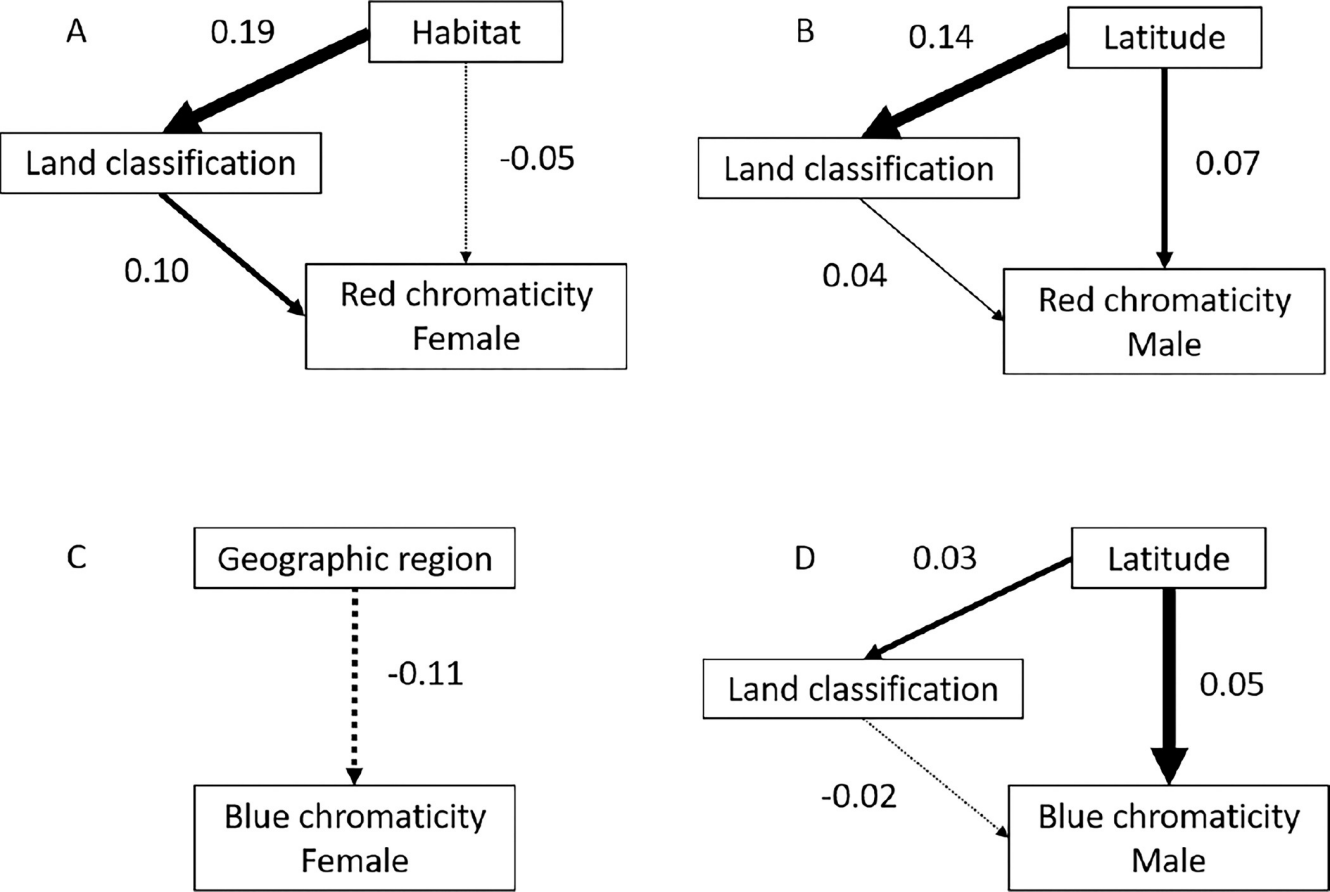
Island plumage color evolution was primarily influenced by ecology and geography. Final path analysis models illustrating the effect of (A) habitat and land classification (island/mainland) on female red chromaticity, (B) geographic region on blue chromaticity, (C) latitude and land classification on male red chromaticity, and (D) male blue chromaticity. Solid lines indicate positive, while dashed lines indicate negative effects.

Our phylogenetic path analyses indicated that plumage color in the Passeriformes is influenced by several factors (S2 Table in S1 File). The top models predicting red chromaticity in females were explained by the direct effects of habitat and land classification and the indirect effect of habitat on land classification (Fig 2, CICc = 451.6), while female blue chromaticity was explained by geographic region (Fig 2, CICc = 506.2). The top model predicting red chromaticity in males was explained by the direct effects of land classification and latitude, and the indirect effect of latitude on land classification (Fig 2, CICc = 399.6). Blue chromaticity in males was explained by the direct effect of diet and land classification and the indirect effect of diet on land classification (Fig 2, CICc = 486.9).

### The island effect varies among Passeriformes families

**Fig 3:**
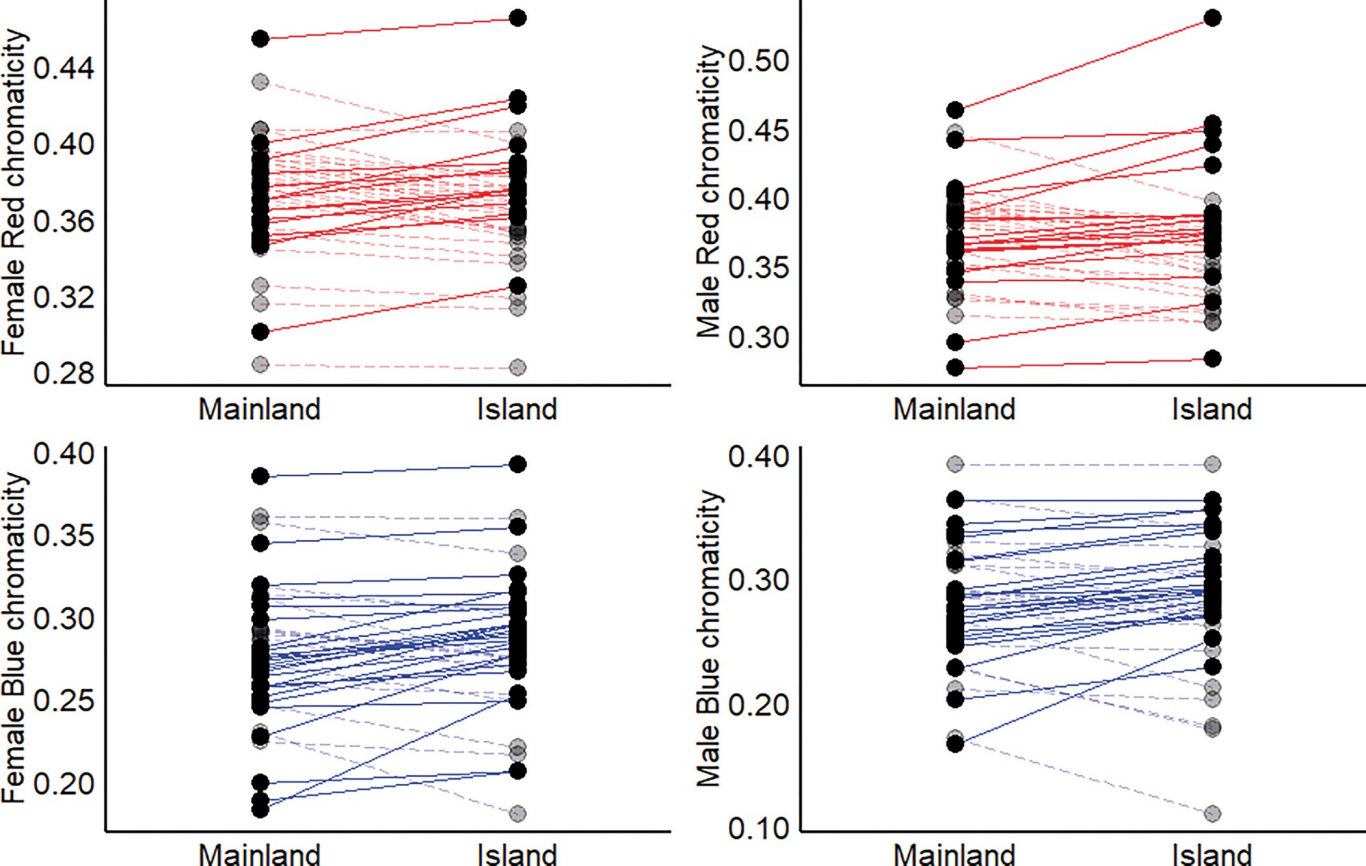
Patterns of island plumage color evolution varied by taxonomic scale. While chromaticity generally varied between mainland and island populations, the direction of this effect varied among passeriform families (dots). Bold dots and solid lines represent increases in red (top panels) and blue chromaticity (bottom panels) and transparent dots and dashed lines indicate decreases. Note axis scales vary to avoid crowding of the lines. Means from raw data are shown.

The direction of color change between mainland and island birds varied among families in the Passeriformes (Fig 3). Red chromaticity increased in island females and males in Fringillidae, Meliphagidae, and Sturnidae but decreased in Estrildidae, Pellorneidae, Pycnonotidae, Tyrannidae, and Vireonidae (S4 Fig in S1 File). Additionally, red chromaticity in females in the Parulidae and Turdidae, and in males in the Muscicapidae and Oriolidae decreased on islands (S4 Fig in S1 File). Blue chromaticity increased in island females and males in Oriolidae, Pellorneidae, Pycnonotidae, Turdidae, Tyrannidae, and Vireonidae but decreased in Sturnidae and Zosteropidae (S4 Fig in S1 File). Blue chromaticity in island females increased in Campephagidae and decreased in Meliphagidae (S4 Fig in S1 File). Male blue chromaticity increased in Muscicapidae and decreased in Fringillidae and Ploceidae between the mainland and islands (S4 Fig in S1 File).

### Island characteristics predict color evolution within island Passeriformes

In our analyses restricted to only island birds, we found the top models for red and blue chromaticity were the reduced models (S3 Table in S1 File) and included island size, island isolation (i.e., distance to the mainland and other islands), and other ecological covariates as above (S4 Table in S1 File). In females, red and blue chromaticity varied with island size, where red was lower and blue was higher on larger islands (S4 Table in S1 File, red: F = 17.64, P<0.0001; blue: F = 12.33, P<0.0001) and by island isolation (S5 Fig in S1 File). Whereas female red chromaticity increased on more distant islands (distance to the mainland: F = 5.69, P = 0.02; distance to other islands: F = 25.35, P<0.0001, S5 Fig in S1 File), blue chromaticity decreased on more isolated islands (S5 Fig in S1 File, distance to other islands: F = 18.84, P<0.0001). We found two-way interactions between latitude and island size and latitude and island isolation, which revealed that red chromaticity increased at higher latitudes regardless of island size (S4 Table in S1 File, F = 10.74, P<0.0001), and on more isolated islands, regardless of latitude (S4 Table in S1 File, latitude x nearest mainland: F = 8.93, P<0.0001; latitude x nearest islands: F = 9.11, P<0.0001). Blue chromaticity decreased on larger islands (S4 Table in S1 File, F = 12.33, P<0.0001) and on more isolated islands (S5 Fig in S1 File, nearest island: F = 18.84, P<0.0001). We also found an interaction between island area and island isolation in blue chromaticity, indicating this plumage feature was lower on more distant islands regardless of island size (S4 Table in S1 File, nearest island: F = 5.24, P = 0.02). Further, there was an interaction between latitude and geographic region (S4 Table in S1 File). While birds in most regions showed increases in red chromaticity and decreases in blue chromaticity, birds in Australasia exhibited decreases in red and increases in blue across latitude (S4 Table in S1 File, F = 4.96, P<0.0001). Additionally, there was an interaction between land classification and diet in female blue chromaticity, indicating this color decreased across latitude in herbivores and increased in invertivores, but not in ominvores (F = 4.21, P = 0.02).

Similarly in males, red and blue chromaticity varied by island size, island isolation, and other ecological covariates (S3 Table in S1 File). Male red chromaticity was lower and blue chromaticity was higher on larger islands (S4 Table in S1 File, red: F = 18.92, P<0.0001, blue: F = 12.37, P<0.0001). In contrast, red was higher, and blue was lower on more isolated islands (S5 Fig in S1 File, red nearest mainland: F = 10.45, P<0.0001; blue nearest mainland: 7.75, P = 0.01; red nearest islands: F = 10.29, P<0.0001; blue nearest islands: F = 12.26, P<0.0001). There were also interactions between island area and distance to the nearest mainland, indicating red chromaticity was higher (F = 10.29, P<0.0001) and blue chromaticity was lower (F = 6.38, P = 0.01) on more distant islands regardless of island size (S4 Table in S1 File). For male blue chromaticity, there was an interaction between island area and geographic region, indicating that although blue chromaticity in male passerines in the Palearctic decreased on larger islands, it increased in males in Afrotropical and Australasian regions (S4 Table in S1 File, F = 3.03, P = 0.02).

## Discussion

Animal coloration is shaped by a variety of ecological and biological pressures, making general patterns of color evolution, especially in an entire order, difficult to disentangle. One factor that may drive plumage evolution is island living, as animal populations on islands may experience unique selection pressures resulting in differences in trait evolution relative to their mainland counterparts. Our phylogenetic comparative analysis revealed plumage coloration of island species differs from their mainland counterparts. However, this pattern is more complex than has been reported previously, is mediated by a number of ecological factors, and varies across taxa. Overall, female passerines on islands exhibited reduced red and enhanced blue coloration but this effect varied among families, with some families showing significant decreases, while others increased in red and blue chromaticity between the mainland and islands. Female and male color variation was also related to ecological covariates, including diet, latitude, habitat, and geographic region. Further, among island species, color variation was affected by island size and isolation. Our results reveal that color evolution is affected by a variety of biological and ecological factors (diet, resource availability, temperature, predation and competition) as well as evolutionary history (family lineages), highlighting the complexity of color evolution in birds. While animal color evolution has been traditionally biased towards males [38], our study reveals interesting patterns regarding the evolution of female communication modalities [39].

The reduced red color of island females suggests a reduction in carotenoid-based coloration. Carotenoid-based coloration is obtained through the consumption, metabolic conversion, and deposition of carotenoid pigments, so our observed reduction in red chromaticity may reflect changes in the diet of island birds. The reduction in red coloration could be attributed to reduced availability of carotenoid precursors in the environment or reflect increased intraspecific competition for sources rich in carotenoid precursors [75]. As an example, when introduced to the Hawaiian Islands, house finches (*Carpodacus mexicanus*), which typically exhibit a red head and breast patches, became orange or yellow soon after being established and carotenoid-restricted diet experiments resulted in the loss of red plumage in male house finches [22]. Alternatively, the decrease in red chromaticity may be a result of relaxed social and/or sexual selection pressure. As islands generally exhibit lower species diversity, the reduction in sympatric species may diminish the necessity of plumage elaboration for species recognition [20]. Our results also revealed an increase in female blue chromaticity on islands. If island birds are carotenoid-deficient, populations may have adapted coloration strategies by shifting endogenous precursors to melanin-based color. One study [20] previously reported that the reduction in plumage brightness in island birds was not associated with increased black colored plumage, such as through status signals like melanin-based badges [76,77], but rather a continuous shift toward duller colors. This shift may be caused by increased melanin or carotenoid content in the feathers, both of which could create thicker keratin cortexes in feathers and reduce the incoherent scattering of light necessary for blue-shifted reflectance [78,79]. Whether island birds are indeed carotenoid deficient is not known; however, supplemental feeding experiments on dull island birds would be a useful study. Further research is also needed to investigate the mechanisms of reduced structural coloration, including microscopy of feather nanostructure. Comparative studies assessing different types of plumage color are needed to disentangle the evolutionary drivers of melanin-, carotenoid-, and structural-based colors of island species [21,80].

One ecological factor that was consistently identified in our analyses as an important predictor of color was latitude. Gloger’s rule predicts lighter colored individuals are found at higher latitudes and darker individuals at lower latitudes [34]. This rule is broadly supported in birds [81]; however, the few comprehensive studies assessing latitudinal effects on color failed to consider the consequences of island habitats or other ecological and biological factors. Our phylogenetic path analyses indicate that the direct and indirect effects of latitude on land classification are the best predictors of color in male Passeriformes. In females, although there was an overall difference in color between island and mainland species, this effect varied by latitude, where red chromaticity in island females was positively related to latitude, while mainland red chromaticity was negatively related to latitude. However, neither land classification or latitude was selected in the top path analysis model for blue chromaticity. One reason latitude may influence color evolution is its link to temperature and precipitation, which may have direct or indirect effects on plumage coloration [47]. Geographic region was the sole predictor of female blue chromaticity in our path analyses, which may be operating similarly to Gloger’s rule. Habitat was another important variable identified in our PGLS models and in the path analysis for female red color. Ambient light may vary among habitats, so selection for crypsis or conspecific signalling may vary given light environment contexts [35].

An interesting result of our study was island living influenced color only in females. Color elaboration in female passerines may be an adaptation to non-migratory life histories [82], which is the case for many island species [15]. Although our results for red chromaticity do not support this notion, blue chromaticity increased on islands supporting the hypothesis that sedentary island life increases at least some aspects of plumage elaboration. Male coloration was not affected by island living, based on our PGLS, but rather was dependent upon other ecological factors such as diet, habitat, and latitude. In island species, island size and the distance to other islands and the mainland were significant as others have found suggesting a reduced role of sexual selection on driving plumage evolution in island species [21]. Limited dispersal to isolated islands may drive plumage color via founder effects, and future work could further assess this hypothesis in species-complexes that have colonized multiple islands [12,32,33].

Macroevolutionary studies are powerful ways to investigate large scale evolutionary patterns; however, they can mask differences at finer taxonomic scales [36,83]. We addressed this issue by analyzing colour evolution at the family-level. Despite finding no overall difference between mainland and island male coloration, several families showed significant increases or decreases (S2 Fig in S1 File). If we had not undertaken the additional family-level analyses, we may have rejected the hypothesis that male color differed between mainland and island species. Similarly, in females we found an overall decrease in red and an increase in blue chromaticity; however, the family-level analyses revealed the direction of these effects varied among families (S2 Fig in S1 File), further highlighting how complex phenotypes, such as plumage color can be affected by different selective forces at different taxonomic scales [84]. Therefore, we join the call to urge future macroevolutionary studies to consider a range of taxonomic scales to elucidate the evolution of phenotypes that are likely being pulled in multiple directions due to differing selective pressures [36]. Identifying the factors that drive some taxa to follow one trajectory while other taxa take a different course should help not only to explain why some species show the ‘island syndrome’ while others do not, but also the complexity of color evolution in continental species. Finally, the strong environmental influence in shaping bird plumage chromaticity on islands from our study suggests a reduced importance of sexual and social factors in driving animal color evolution. Why sexual factors should play a lesser role on islands is not clear, but highlights the need for experimental studies on the patterns of social and sexual selection in island birds compared to their continental counterparts.

## Supporting information

S1 Dataset Excel spreadsheet of all data used for analyses. Metadata, with explanation of each column, are included in first tab on spreadsheet.
(XLSX)

S1 File Supporting tables (S1-4) and figures (S1-S5). (DOCX)
