# Supplementary material for: Global patterns of plumage color evolution in island-living passeriform birds
Source: PLoS One. 2023 Dec 15;18(12):e0294338. doi: 10.1371/journal.pone.0294338 (PMC10723677; doi:10.1371/journal.pone.0294338)
Supplement: S1 File — (DOCX) [file pone.0294338.s002.docx]

**Supporting information**

**Supporting dataset.**

Excel spreadsheet of all data used for analyses. Metadata, with explanation of each column, are included in first tab on spreadsheet.

**Table S1. PGLS Model Selection.**

Full and reduced model selection results to assess the relationships between island and mainland classification, biological, and ecological variables on red and blue chromaticity in female and male Passeriformes birds.

| Sex | Variable | Model | K | AICc | ΔAICc | Likelihood | AICcWt | LL | Cum.Wt |
| --- | --- | --- | --- | --- | --- | --- | --- | --- | --- |
| Female | Red chromaticity | Reduced | 26 | -18362.5 | 0 | 1 | 0.72 | 9207.40 | 0.72 |
|  |  | Full | 27 | -18360.6 | 1.88^1^ | 0.39 | 0.28 | 9207.47 | 1 |
|  | Blue chromaticity | Reduced | 24 | -15856.7 | 0 | 1 | 0.59 | 7952.50 | 0.59 |
|  |  | Full | 27 | -15856 | 0.73^2^ | 0.69 | 0.41 | 7955.16 | 1 |
| Male | Red chromaticity | Reduced | 24 | -16349 | 0 | 1 | 0.77 | 8198.64 | 0.77 |
|  |  | Full | 27 | -16346.6 | 2.40^3^ | 0.30 | 0.23 | 8200.47 | 1 |
|  | Blue chromaticity | Reduced | 23 | -14604.9 | 0 | 1 | 0.91 | 7325.58 | 0.91 |
|  |  | Full | 27 | -14600.3 | 4.59 | 0.10 | 0.09 | 7327.33 | 1 |

ΔAICc<4 model comparison results: ^1^log ratio=0.14, P=0.7; ^2^log ratio=5.33, P=0.15; ^3^log ratio=3.66, P=0.3, indicating reduced models outperform full models. K=number of parameters, Likelihood=model likelihood, LL=log likelihood, Cum.Wt=cumulative model weight.

**Table S2.** **Island plumage color evolution was primarily influenced by ecology and geography based on Phylogenetic Path Analyses.**

Phylogenetic Path Analysis model selection results testing the effect of biological and ecological variables on Passeriformes island and mainland species colour. The top model appears in bold.

|  | Model | k | q | C | CICc | ΔCICc | L | w |
| --- | --- | --- | --- | --- | --- | --- | --- | --- |
| Female Red Chromaticity | **Land class+habitat+(land class~habitat)** | **12** | **9** | **433.6** | **451.61** | **0.00** | **1** | **1** |
|  | Region | 14 | 7 | 486.7 | 500.71 | 49.10 | 2.17E-11 | 2.17E-11 |
|  | Land class+diet+(land class~diet) | 12 | 9 | 519.1 | 537.17 | 85.56 | 2.64E-19 | 2.64E-19 |
|  | Land class | 14 | 7 | 527.9 | 541.92 | 90.31 | 2.45E-20 | 2.45E-20 |
|  | Habitat+diet+(habitat~diet) | 12 | 9 | 558.0 | 576.05 | 124.44 | 9.50E-28 | 9.50E-28 |
|  | Habitat | 14 | 7 | 565.9 | 579.88 | 128.27 | 1.40E-28 | 1.40E-28 |
|  | Diet | 14 | 7 | 566.5 | 580.52 | 128.91 | 1.02E-28 | 1.02E-28 |
|  | Latitude | 14 | 7 | 568.3 | 582.36 | 130.75 | 4.06E-29 | 4.06E-29 |
| Female Blue chromaticity | **Region** | **14** | **7** | **492.2** | **506.19** | **0.00** | **1** | **1** |
|  | Land class+diet+(land class~diet) | 12 | 9 | 524.9 | 542.92 | 36.73 | 1.06E-08 | 1.06E-08 |
|  | Habitat+diet+(habitat~diet) | 12 | 9 | 527.2 | 545.23 | 39.04 | 3.33E-09 | 3.33E-09 |
|  | Habitat | 14 | 7 | 533.2 | 547.20 | 41.01 | 1.25E-09 | 1.25E-09 |
|  | Land class | 14 | 7 | 533.2 | 547.25 | 41.06 | 1.21E-09 | 1.21E-09 |
|  | Diet | 14 | 7 | 549.3 | 563.35 | 57.16 | 3.86E-13 | 3.86E-13 |
|  | Latitude | 14 | 7 | 549.8 | 563.84 | 57.65 | 3.03E-13 | 3.03E-13 |
| Male Red Chromaticity | **Land class+latitude+(land class~latitude)** | **12** | **9** | **381.6** | **399.64** | **0.00** | **1** | **1** |
|  | Latitude | 14 | 7 | 466.8 | 480.82 | 81.18 | 2.35E-18 | 2.35E-18 |
|  | Land class+diet+(land class~diet) | 12 | 9 | 472.6 | 490.69 | 91.05 | 1.70E-20 | 1.70E-20 |
|  | Land | 14 | 7 | 483.0 | 497.02 | 97.38 | 7.15E-22 | 7.15E-22 |
|  | Region | 14 | 7 | 489.7 | 503.71 | 104.07 | 2.52E-23 | 2.52E-23 |
|  | Habitat+diet+(habitat~diet) | 12 | 9 | 499.2 | 517.25 | 117.61 | 2.89E-26 | 2.89E-26 |
|  | Diet | 14 | 7 | 503.9 | 517.96 | 118.32 | 2.02E-26 | 2.02E-26 |
|  | Habitat | 14 | 7 | 506.4 | 520.46 | 120.82 | 5.81E-27 | 5.81E-27 |
| Male Blue Chromaticity | **Land class+diet+(land class~diet)** | **12** | **9** | **468.8** | **486.85** | **0.00** | **1.00** | **0.51** |
|  | Latitude | 14 | 7 | 474.5 | 488.52 | 1.68 | 0.43 | 0.22 |
|  | Region | 14 | 7 | 475.5 | 489.49 | 2.64 | 0.27 | 0.14 |
|  | Habitat+diet+(habitat~diet) | 12 | 9 | 471.8 | 489.80 | 2.95 | 0.23 | 0.12 |
|  | Diet | 14 | 7 | 478.9 | 492.96 | 6.11 | 0.05 | 0.02 |
|  | Land class | 14 | 7 | 486.2 | 500.24 | 13.39 | 0.00 | 0.00 |
|  | Habitat | 14 | 7 | 487.0 | 501.00 | 14.15 | 0.00 | 0.00 |

**Table S3.** **Island-dwelling bird PGLS model selection.**

Full and reduced model selection results to assess the relationships between classification, biological, and ecological variables on red and blue chromaticity in female and male Passeriformes island birds.

| Sex | Variable | Model | K | AICc | ΔAICc | LL | AICcWt | LL | Cum.Wt |
| --- | --- | --- | --- | --- | --- | --- | --- | --- | --- |
| Female | Red chromaticity | Reduced | 29 | -1880.72 | 0 | 1 | 1.00 | 970.51 | 1.00 |
|  |  | Full | 37 | -1868.86 | 11.86 | 0.00 | 0.00 | 973.31 | 1 |
|  | Blue chromaticity | Reduced | 27 | -1463.26 | 0 | 1 | 1.00 | 759.63 | 1.00 |
|  |  | Full | 37 | -1446.89 | 16.38 | 0.00 | 0.00 | 762.32 | 1 |
| Male | Red chromaticity | Reduced | 26 | -1665.93 | 0 | 1 | 1.00 | 859.89 | 1.00 |
|  |  | Full | 37 | -1649.28 | 16.65 | 0.00 | 0.00 | 863.52 | 1 |
|  | Blue chromaticity | Reduced | 27 | -1338.81 | 0 | 1 | 1.00 | 697.40 | 1.00 |
|  |  | Full | 37 | -1322.6 | 16.21 | 0.00 | 0.00 | 700.18 | 1 |

K=number of parameters, Likelihood=model likelihood, LL=log likelihood, Cum.Wt=cumulative model weight.

**Table S4.** **Island-dwelling plumage colour evolution was affected by island geography, habitat, and diet.**

AIC selected model results demonstrating the effect of fixed effects of biological, ecological, and island characteristics on island dwelling female and male Passeriformes plumage coloration.

| Sex | Variable | Fixed effect | *df* | *F* | *P* |
| --- | --- | --- | --- | --- | --- |
| Female | Red chromaticity | **Habitat** | **3** | **7.07** | **<0.0001** |
|  |  | Diet | 2 | 1.61 | 0.20 |
|  |  | **Geographic region** | **4** | **3.75** | **0.01** |
|  |  | Latitude | 1 | 0.84 | 0.36 |
|  |  | **Island size** | **1** | **17.64** | **<0.0001** |
|  |  | **Nearest mainland** | **1** | **5.69** | **0.02** |
|  |  | **Nearest Island group** | **1** | **25.35** | **<0.0001** |
|  |  | **Diet x Latitude** | **2** | **4.59** | **0.01** |
|  |  | **Geographic region x latitude** | **4** | **4.03** | **<0.0001** |
|  |  | **Latitude x islands size** | **1** | **10.74** | **<0.0001** |
|  |  | **Latitude x Nearest mainland** | **1** | **8.93** | **<0.0001** |
|  |  | **Latitude x nearest island group** | **1** | **9.11** | **<0.0001** |
|  |  | Geographic region x island size | 4 | 1.04 | 0.39 |
|  |  | **Island size x nearest mainland** | **1** | **6.10** | **0.01** |
|  | Blue chromaticity | **Habitat** | **3** | **5.89** | **<0.0001** |
|  |  | Diet | 2 | 1.68 | 0.19 |
|  |  | Geographic region | 4 | 2.19 | 0.07 |
|  |  | Latitude | 1 | 0.15 | 0.69 |
|  |  | **Island size** | **1** | **12.33** | **<0.0001** |
|  |  | Nearest mainland | 1 | 2.98 | 0.08 |
|  |  | **Nearest Island group** | **1** | **18.84** | **<0.0001** |
|  |  | **Diet x Latitude** | **2** | **4.21** | **0.02** |
|  |  | **Geographic region x latitude** | **4** | **4.96** | **<0.0001** |
|  |  | Latitude x Nearest mainland | 1 | 2.84 | 0.09 |
|  |  | **Geographic region x island size** | **4** | **5.13** | **<0.0001** |
|  |  | **Island size x nearest mainland** | **1** | **5.24** | **0.02** |
| Male | Red chromaticity | **Habitat** | **3** | **2.95** | **0.03** |
|  |  | Diet | 2 | 0.48 | 0.62 |
|  |  | **Geographic region** | **4** | **3.82** | **<0.0001** |
|  |  | Latitude | 1 | 1.04 | 0.31 |
|  |  | **Island size** | **1** | **18.92** | **<0.0001** |
|  |  | **Nearest mainland** | **1** | **10.45** | **<0.0001** |
|  |  | **Nearest island group** | **1** | **10.29** | **<0.0001** |
|  |  | Diet x Latitude | 2 | 1.72 | 0.18 |
|  |  | **Geographic region x latitude** | **4** | **8.24** | **<0.0001** |
|  |  | Geographic region x island size | 4 | 2.28 | 0.06 |
|  |  | **Island size x nearest mainland** | **1** | **10.29** | **<0.0001** |
|  | Blue chromaticity | **Habitat** | **3** | **3.76** | **0.01** |
|  |  | Diet | 2 | 2.26 | 0.11 |
|  |  | **Geographic region** | **4** | **3.11** | **0.01** |
|  |  | Latitude | 1 | 0.80 | 0.37 |
|  |  | **Island size** | **1** | **12.37** | **<0.0001** |
|  |  | **Nearest mainland** | **1** | **7.75** | **0.01** |
|  |  | **Nearest Island group** | **1** | **12.26** | **<0.0001** |
|  |  | Diet x Latitude | 2 | 2.31 | 0.10 |
|  |  | **Geographic region x latitude** | **4** | **6.70** | **<0.0001** |
|  |  | Latitude x Nearest island group | 1 | 0.49 | 0.48 |
|  |  | **Geographic region x island size** | **4** | **3.03** | **0.02** |
|  |  | **Island size x nearest mainland** | **1** | **6.38** | **0.01** |

**
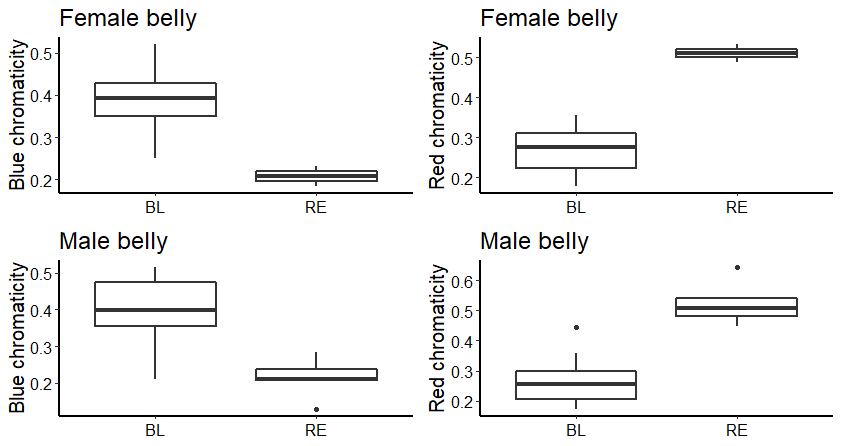
**

**Fig S1.** **Chromaticity captures red and blue plumage colour variation.**

Female and male blue (left panels) and red chromaticity (right panels) for Thraupidae belly plumage patches classified as blue (“BL”) or red (“RE”) by an independent observer. There is little overlap in the distribution of chromaticity values for patches classified as red or blue, suggesting that chromaticity effectively captures the variation in red and blue plumage colouration.


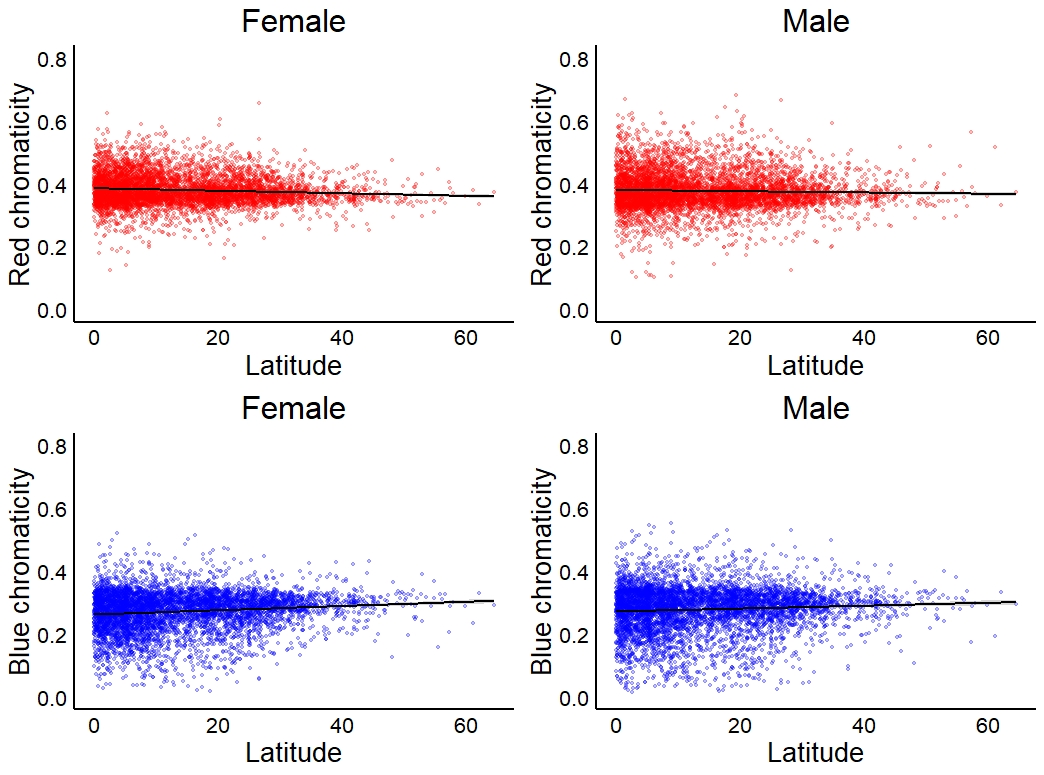


**Fig S2.** **Passeriformes plumage colour varies by latitude.**

Female and male colour variation of passerine birds (n=5,693) across latitude. Top panels: Red chromaticity was significantly higher at lower latitudes for females (F=65.4, P<0.0001) and males (F=15.8, P<0.0001). Bottom panels: Blue chromaticity was positively related to latitude in females (F=75.6, P<0.0001) and males (F=14.01, P<0.0001). Figures show raw data.


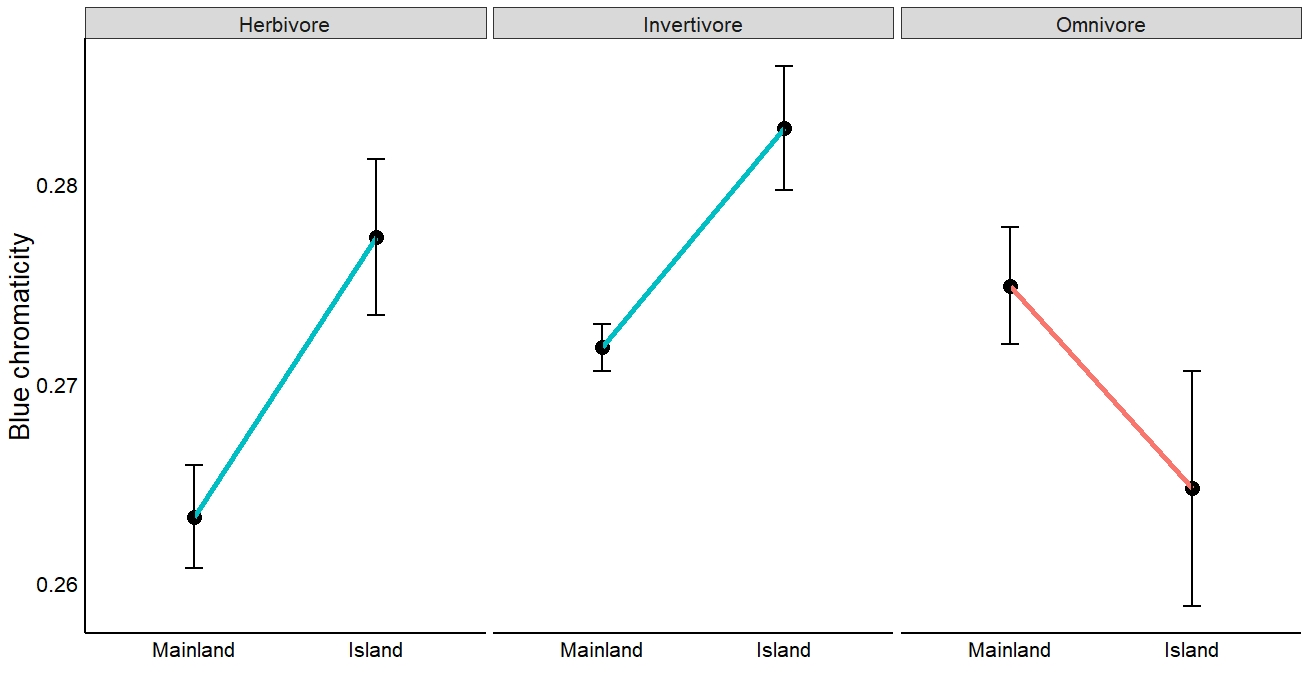


**Fig S3.** **Passeriformes plumage colour varies by diet classification.**

Female blue chromaticity (±SE) of passerine birds (n=5,693) of varying diets on islands and mainlands. The interaction between land type and diet indicated omnivore blue chromaticity did not vary between island and mainlands, but blue chromaticity was lower on mainland systems in herbivores and invertivores (F=17.93, P<0.0001). Figures show raw data.


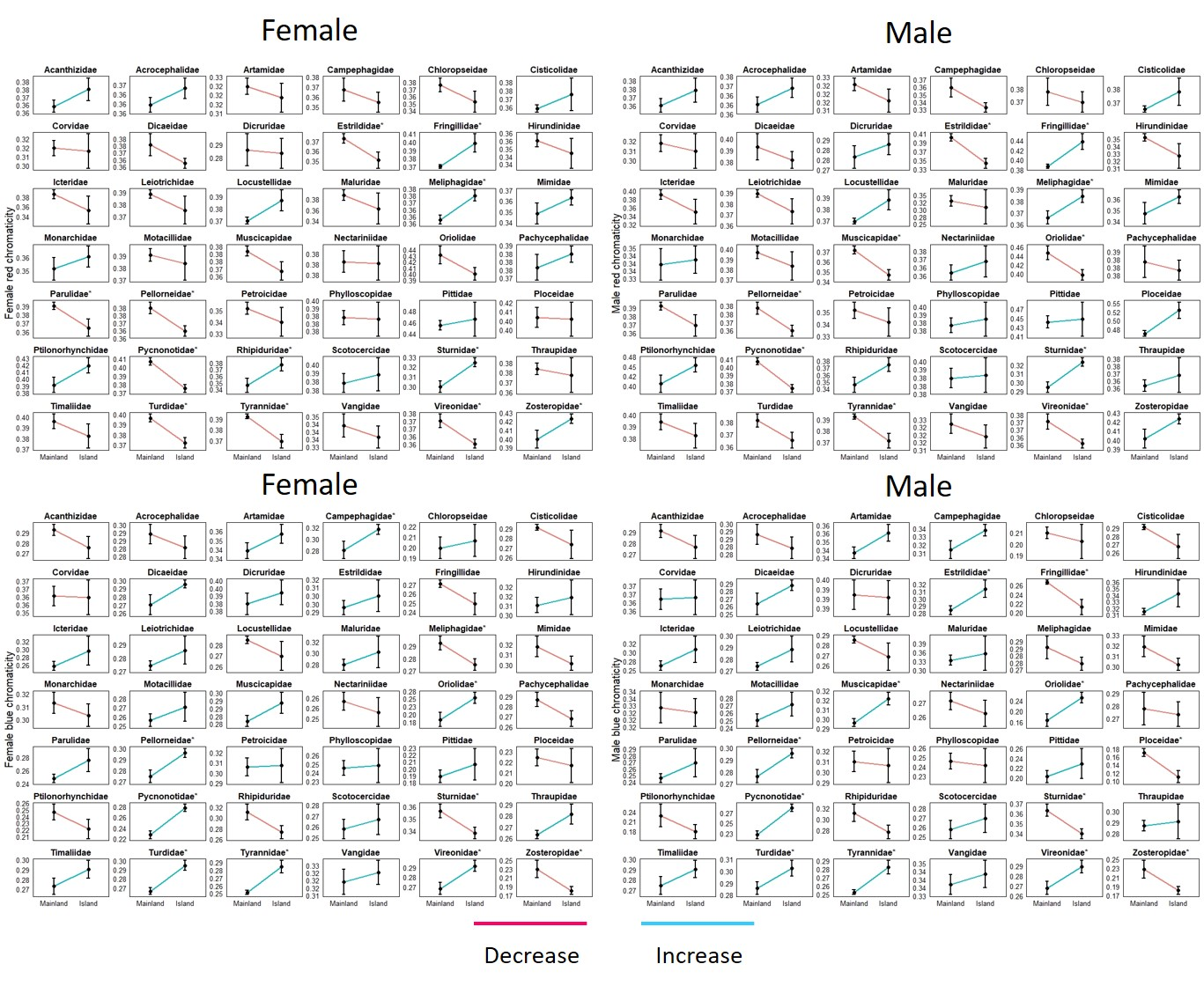


**Fig S4.** **Patterns of colour differences between island and mainland passeriform birds varied by taxonomic scale.**

The island effect on colour evolution varied among Passeriformes families. Top panels: Red chromaticity; bottom panels: blue chromaticity. Red lines indicate decreases in chromaticity, while blue lines indicate increases in chromaticity between mainland and islands. Asterisks following family name indicate significant differences between mainlands and islands. Figures show raw data.


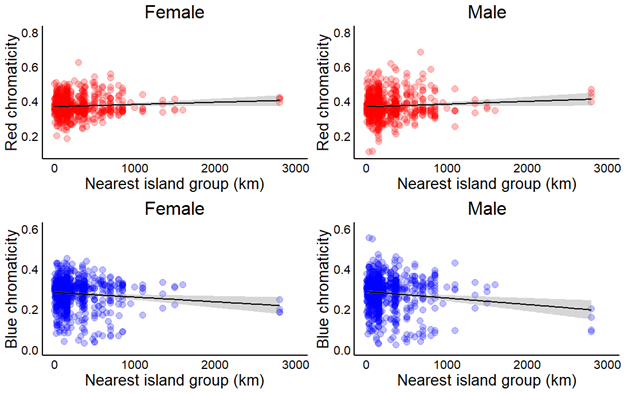


**Fig S5.** **In island-dwelling passeriforms, plumage colour was affected by island isolation.**

The relationship between passerine female and male chromaticity (n=1,183) and nearest island group (km). In females (F=25.4, P<0.0001) and males (F=10.3, P<0.0001), red chromaticity increased (top panels) and (bottom panels) blue chromaticity decreased (females: F=18.8, P<0.0001; males: F=12.3, P<0.0001) on more isolated islands. Raw data are presented.
